# Lymphatic-immune interactions in the musculoskeletal system

**DOI:** 10.3389/fimmu.2025.1578847

**Published:** 2025-05-27

**Authors:** Hanyu Liu, Lu Liu, Yuheng Zhang, Qi Tian, Zhangfan Ding, Junyu Chen, Anjali P. Kusumbe

**Affiliations:** ^1^ Tissue and Tumor Microenvironments Lab, Lee Kong Chian School of Medicine, Nanyang Technological University, Singapore, Singapore; ^2^ State Key Laboratory of Oral Diseases, National Center for Stomatology, National Clinical Research Center for Oral Diseases, Department of Head and Neck Oncology, West China Hospital of Stomatology, Sichuan University, Chengdu, China; ^3^ State Key Laboratory of Oral Diseases, National Center for Stomatology, National Clinical Research Center for Oral Diseases, Department of Prosthodontics, West China Hospital of Stomatology, Sichuan University, Chengdu, China; ^4^ University of Coimbra, Multidisciplinary Institute of Ageing (MIA-Portugal), Coimbra, Portugal

**Keywords:** lymphatics, immune, bone, aging, musculoskeletal

## Abstract

Traditionally, the role of lymphatic vessels has been understood as primarily involving fluid transport and immune surveillance. In addition to these roles, recent studies have revealed a paracrine function of lymphatics through the dissemination of inductive factors, known as lymphangiocrine signals including in musculoskeletal physiology and diseases. These signals play diverse roles, including maintaining tissue equilibrium and facilitating regeneration. Impaired lymphangiocrine signaling and lymphatic function are features of musculoskeletal diseases. This review summarizes dysregulation of lymphatic vessels and interactions with immune cells during musculoskeletal diseases. Further, this review provides insights into lymphangiocrine signals as a potential therapeutic target.

## Introduction

**Figure 1:**
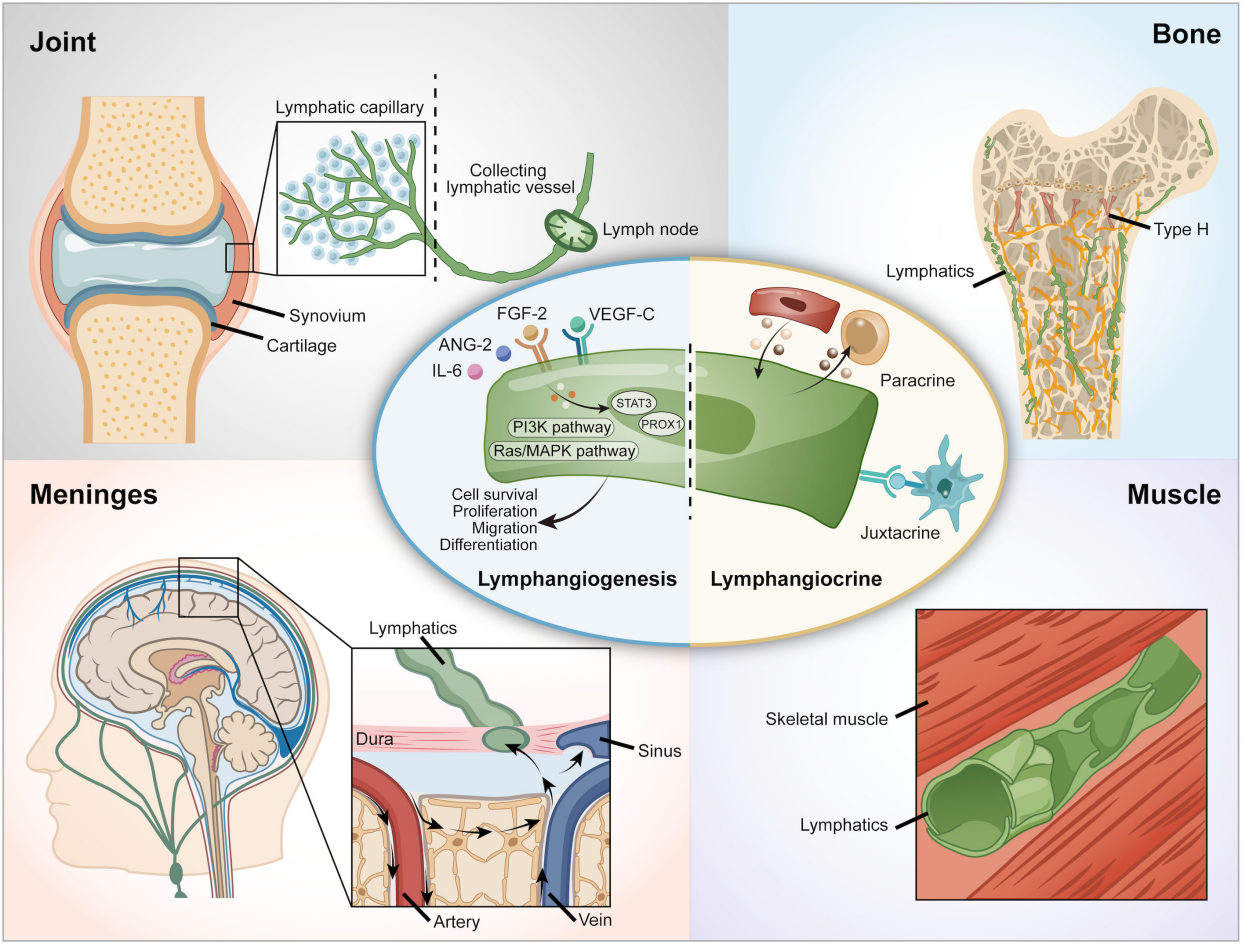
Lymphatic vessels in various organs. VEGF-C, Vascular Endothelial Growth Factor - C; VEGFR-3, Vascular Endothelial Growth Factor Receptor-3.

The lymphatic system is a fundamental pillar of human physiology, forming an extensive network of vessels, nodes, and organs that orchestrate fluid balance, immune surveillance, and tissue homeostasis (1–3). Lymphatic vessels, lined by specialized lymphatic endothelial cells (LECs), exhibit remarkable structural and functional diversity shaped by their local tissue environments (**Figure 1**). While the lymphatic vasculature of the skin and gut—central to immune monitoring and lipid absorption—is well-mapped, the lymphatic networks within the musculoskeletal system remain largely unexplored. Yet, emerging evidence highlights their indispensable role in tissue repair, inflammation resolution, and maintaining skeletal integrity (2, 4, 5). Understanding the unique biology of the musculoskeletal lymphatics offers a compelling opportunity to uncover new mechanisms of tissue regeneration and develop targeted therapies for musculoskeletal disorders. Lymphangiogenesis occurs during regeneration and inflammation (6, 7), when lymphatics remodel and expand new vessels supporting fluid drainage and immune cell trafficking. Vascular endothelial growth factor C (VEGF-C) is the key modulator in regulation of lymphatic endothelial cell proliferation and migration (8). Some proinflammatory cytokines such as TNFα and IL-1 can also induce lymphangiogenesis during inflammation.

Beyond the traditional draining function, recent research has found a novel function of lymphatic vessels. Lymphatic vessels secrete a variety of factors, including growth factors, extracellular matrix components, cytokines, and chemokines, which communicate with other tissues through paracrine or juxtacrine signaling mechanisms. This phenomenon is referred to as ‘lymphangiocrine’ or ‘angiocrine signaling’ (9, 10) (**Figure 1**). For example, lymphatic vessels support heart development, homeostasis and regeneration following an injury via Reelin, which is a lymphatic endothelial cell-derived protein (11). In the intestine, lymphatic vessels crosstalk with intestinal crypts via Rspo3 and Reelin to help recovery from cytotoxicity (12, 13). In the bone, the lymphangiocrine factor CXCL12 mediates the interactions between lymphatic endothelial cells and hematopoietic stem cells (HSCs) and bone (9). Since the discovery of the presence and importance of lymphatic vessels in bone, attention to the role of lymphangiocrine signaling has arisen. However, an overview of lymphatic vessels in skeletal tissues has not been studied so far.

Aging is characterized by a gradual decline in physiological function, leading to increased vulnerability to various diseases across multiple organ systems, including the lymphatic vessels (14). This decline is a significant factor in the onset of age-related diseases, with the most prevalent being neurodegenerative disorders, cancer, cardiovascular diseases, and musculoskeletal conditions (15). Among these, the elderly are particularly susceptible to injuries and degenerative disorders of the musculoskeletal system, including sarcopenia and osteoarthritis (OA). These musculoskeletal disorders have a considerable economic impact (16). Despite this, the specific mechanisms by which aging affects lymphatic vessel function and disrupts lymphangiocrine signaling within the musculoskeletal system remain largely unexplored.

Since clinical efforts to target lymphangiogenic growth factors for skeletal diseases have not been entirely successful, further research is needed to identify new targets. Thus, lymphangiocrine signaling needs investigation as a potential therapeutic target. In this review, we will highlight the traditional and new roles of lymphatic vessels in bone and other musculoskeletal tissues. Moreover, this review will discuss the dysregulation of lymphatic circulation and lymphangiocrine signaling associated with the musculoskeletal diseases. Furthermore, we indicate potential approaches for pharmacological targeting of the lymphatic vessels for treating these diseases. Lastly, we address the existing gaps in our understanding and offer insights into future research directions in this field.

## Lymphatic vessels across musculoskeletal tissues

### Joints and skeletal muscle

Lymphatic vessels and their capillaries are intricately distributed within the soft tissues surrounding the articular spaces in joints, playing a crucial role in the removal of harmful molecules (17). Immunolocalization studies have confirmed that lymphatic vessels, along with macrophages, are integral to clearing metabolic waste and extracellular matrix breakdown products that arise from the rapid remodeling of knee joint tissues during development (18).

The lymphatics in skeletal muscle exhibit several distinct characteristics. Notably, skeletal muscle lymphatics are devoid of smooth muscle within their walls (19), rendering them non-contractile, unlike lymphatics found in other tissues, such as the mesentery. Histological analyses reveal a close association between lymphatic vessels and the accompanying arterioles and venules in skeletal muscle. This close proximity to muscle fibers facilitates the dynamic opening and closing of lymphatics, regulated primarily by the periodic vasomotion of arterioles and the contraction of surrounding muscle tissue (20). For instance, during the contraction of arterioles or muscle fibers, lymphatic vessels become more permeable and open; conversely, they tend to close when arterioles are relaxed. Consequently, the formation of lymph fluid is directly influenced by muscle activity, such as during exercise, which significantly increases arterial blood flow to skeletal muscle (21).

The impact of muscle pathologies, such as muscular dystrophy, on muscle lymphatics remains poorly understood. Research has indicated that following hindlimb unloading, there is a reduction in lymphatic capillary density, accompanied by diminished mRNA levels of VEGF-C and VEGF-D (22). In cases of Duchenne Muscular Dystrophy, where abnormalities in the dystrophin gene and protein occur, alterations in the spleen and its associated lymphatic vessels have also been noted (23, 24). Furthermore, lymphatic vessels are involved in the recovery processes following muscle injuries. Morphological changes in intramuscular lymphatic vessels have been observed at inflammatory sites during the initial phases of muscle recovery (25). This suggests that abnormal lymphatic drainage may exacerbate inflammation and immune dysregulation, further complicating the pathology of skeletal muscle and potentially other tissues. To enhance our understanding of muscle pathologies, further research should also address the paracrine functions of lymphatic vessels in muscle tissue, as these functions may reveal additional insights into their roles in health and disease.

### Bone

Recent research on bone vascularization has yielded conflicting results regarding the presence and functional significance of lymphatic vessels in bone tissue. Historical studies reported that Indian ink could migrate to lymph nodes after being injected into long bones, and high-molecular-weight compounds like ferritin were found to reach the bone’s periosteal surface following administration into the bone marrow (26–28). These findings suggested a potential connection between the bone marrow and the bone surface; however, these studies struggled to distinguish lymphatic vessels from blood vessels due to the lack of specific lymphatic endothelial cell markers.

Currently, several markers such as LYVE1, PROX1, and PDPN are routinely employed for the identification and characterization of lymphatic endothelial cells in various tissues (29). Despite these advancements, traditional 2D immunohistochemical staining methods have not successfully visualized lymphatic vessels within bone (30). Some studies suggest that lymphatic vessels may be largely absent in bone and only appear under pathological conditions such as Gorham-Stout Disease (30–33). These limitations in detection likely arise from the potential sparsity and uneven distribution of lymphatic vessels within healthy bone.

Previous studies have focused on the anatomy and function of vertebral lymphatic vessels in the vertebral canal (31). The choice of tissue clearing protocols and sample preparation techniques significantly affects antibody penetration and deep imaging efficacy. Most current imaging methods for whole bones rely on genetically modified reporter mice, and while methods like PEGASOS are useful for immunostaining, they typically produce low-resolution data (31, 33–35). However, recent advances in 3D light-sheet imaging have successfully revealed the presence of lymphatic vessels in bones (9), significantly enhancing our understanding of bone lymphatics and potentially leading to novel therapeutic strategies.

**Figure 2:**
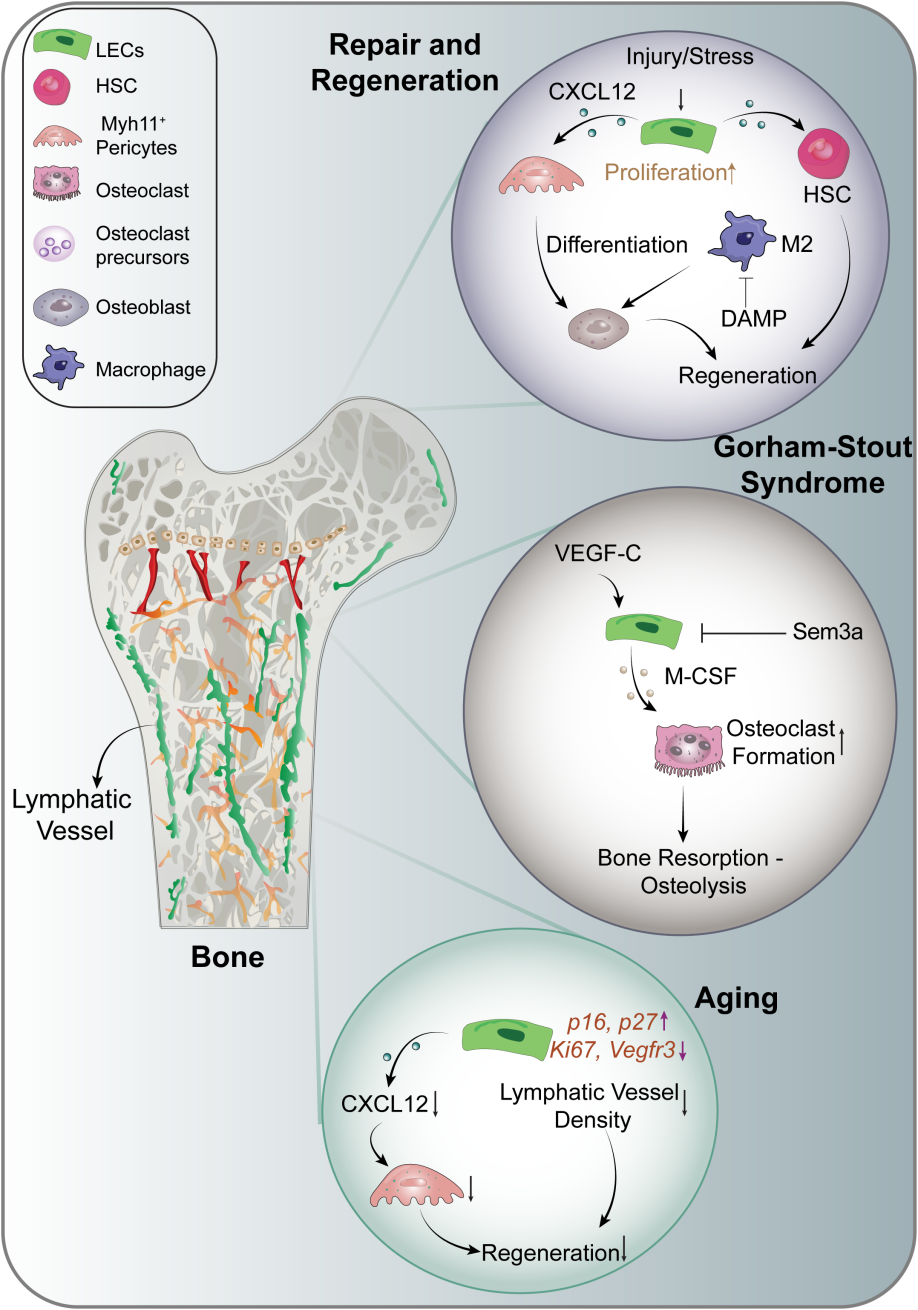
Lymphangiocrine signaling in the musculoskeletal system. CXCL12, C-X-C Motif Chemokine Ligand 12; DAMP, Damage- Associated Molecular Patterns; HSC, Hematopoietic Stem Cells; IL-6, Interleukin 6; M-CSF, Macrophage Colony Stimulating Factor; Sem3a, Semaphorin-3a.

This breakthrough also underscores the role of lymphatic endothelial cells in bone regeneration (**Figure 2**). In mouse models, VEGF-C and IL-6, are essential for lymphangiogenesis and the regeneration of hematopoietic stem cells (HSCs) following genotoxic stress (9). Additionally, CXCL12 derived from lymphatic endothelial cells has been shown to facilitate angiogenesis by supporting HSC regeneration and promoting the expansion of Myh11-positive pericytes. The absence of CXCL12 can lead to reduced bone marrow cellularity and impaired hematopoietic reconstitution, ultimately resulting in decreased bone mass and reduced expression of osteogenesis markers following irradiation (9). These findings suggest that targeting bone lymphatics could be a potential therapeutic strategy to stimulate hematopoietic and bone regeneration, particularly in conditions of stress and injury, as well as for age-related impairments in repair and regeneration (9). Furthermore, these insights hold significant implications for bone diseases and metastasis.

Lymphatic vessels have been identified in multiple murine bones, including the sternum, vertebrae, ribs, femur, skull, and hip bone (9). The discovery of lymphatics in the meninges (36) further raises the possibility of a functional link between skull bone lymphatics and meningeal lymphatic networks. However, the exact drainage pathways of bone-associated lymphatics remain incompletely defined. It is still unclear whether these vessels directly connect to regional lymph nodes. Early tracer studies, in which dyes injected into long bones migrated to lymph nodes, suggest a potential drainage connection (26–28). To resolve these uncertainties, future work must focus on high-resolution mapping of lymphatic drainage routes within bone to clarify their anatomical connections and functional relevance to the broader lymphatic system.

Lymphatic vessels play a crucial role in the musculoskeletal system, aiding in the removal of excess fluid, clearing waste products, and supporting immune responses (1). During inflammation, lymphatics respond by collecting interstitial fluid from peripheral regions, encompassing inflamed tissues (2). They transport this fluid to lymph nodes (LNs) and subsequently return it to the bloodstream through the intricate network of lymphatic vessels (3). Interstitial fluid carries a diverse array of molecules, including soluble foreign antigens, tissue-specific self-antigens, and cellular components (37, 38).

Osteocytes, located within lacunae in the mineralized bone matrix, can indirectly influence other organs through their endocrine functions (39). They are connected to both the bone surface and vasculature, and the canalicular fluid that flows through the lacunocanalicular space is essential for preserving osteocyte viability through mechanical stimuli (39). This fluid flow is a crucial factor in osteocyte physiology and survival (40, 41). The identification of lymphatic vessels in bone raises questions about their traditional roles in draining excess fluid and clearing waste products from tissue. Current literature indicates that in healthy bone, fluid flows from the bone marrow cavity to the periosteal lymphatic vessels via Volkmann and Haversian canals (42). A recent study explored the contributions of lymphatic vessel drainage to fracture repair, revealing that lymphatic platelet thrombosis can impede healing by limiting lymphatic fluid flow (43). This investigation showed that promoting lymphatic drainage leads to reduced neutrophil counts and an increase in beneficial immune cells, such as M2-like macrophages in injury areas, which is crucial for osteoblast survival and bone healing (43). Lymphatics facilitate this process by effectively transporting damage-associated molecular patterns essential for the healing response (43). These findings open new avenues for improving bone fracture treatments, while further studies are necessary to fully understand the role of lymphatic vessel drainage in bone. Additionally, the interaction between canalicular fluid and lymphatic vessels, as well as the role of osteocytes in this relationship, warrants further investigation.

### Skull-associated meningeal lymphatic vessels

Recent advances in neuroscience have illuminated the critical role of skull-associated meningeal lymphatic vessels (mLVs) in central nervous system (CNS) physiology and pathology (36). These conventional lymphatic vessels, located within the dura mater and aligned with the dural venous sinuses and meningeal arteries of the skull, serve as key conduits for draining cerebrospinal fluid (CSF), interstitial fluid, and immune cells from the CNS into deep cervical lymph nodes, maintaining CNS homeostasis and facilitating immune surveillance (36, 44). At the cellular level, mLVs mediate the trafficking of immune cells—including T cells, B cells, and antigen-presenting cells—out of the CNS, a process critical for initiating immune responses and preserving immune tolerance. Disruption of this skull-based drainage system is increasingly linked to neuroinflammation and the pathogenesis of neurological diseases, including Alzheimer’s disease, where impaired mLV function contributes to amyloid-beta accumulation (44, 45). Lymphangiogenic signaling, particularly via VEGF-C, has been shown to promote mLV development and function. Therapeutic strategies leveraging VEGF-C delivery have demonstrated efficacy in enhancing lymphatic clearance, reducing neuroinflammatory markers, and improving functional outcomes in models of stroke and neurodegeneration (46, 47). These discoveries underscore the central role of skull meningeal lymphatics in neuroimmune communication and offer promising cellular and molecular targets for therapeutic intervention in CNS disorders.

## Dysregulated lymphatic vessels in aging

With aging, persistent low-grade inflammation, termed “inflammaging,” disrupts the delicate equilibrium of bone remodeling, resulting in heightened bone loss. This phenomenon arises from several interconnected mechanisms. First, immune cells increase the secretion of pro-inflammatory cytokines, such as interleukin-1 beta (IL-1β) and tumor necrosis factor-alpha (TNFα), which induce excessive osteoclast activity responsible for bone resorption. Second, osteoblasts, which are critical for new bone formation, exhibit decreased functionality with age, further exacerbating the shift toward bone loss. Third, the intricate communication between bone cells and immune cells becomes dysregulated, resulting in dysfunctional bone remodeling. Collectively, these age-related inflammatory processes lead to a decline in bone mineral density and an increased susceptibility to fractures, thus elucidating the relationship between inflammation and bone health in older individuals (48).

Lymphangiogenesis is closely associated with inflammation in musculoskeletal tissues. For example, inflammatory triggers such as TNFα and IL-1 induce lymphangiogenesis by promoting the production of VEGF-C from immune cells, which plays a pivotal role in stimulating lymphatic formation (4, 49). Once the inflammatory stimulus is resolved, lymphatic endothelial cells can revert to a quiescent state, allowing lymphatic vessels to restore homeostasis.

However, age is associated with diminished lymphatic drainage function. Aged lymphatic vessels exhibit lower contractile pressures and reduced pumping frequency, impairing their effectiveness in fluid clearance (14, 50). Furthermore, morphological changes in aged lymphatic vessels include looser cell-cell junctions and a reduction in the glycocalyx, which results in lymphatic vessel leakage. The decline in lymphatic drainage capacity may contribute to the accumulation of toxic waste in musculoskeletal tissues, exacerbating various aging related disorders (51, 52).

Aging is linked with dysregulated lymphangiocrine signaling, adversely affecting the function of lymphatic endothelial cells (9, 45, 50). Age-related chronic inflammation disrupts the interaction between lymphatic vessels and immune cells, which is crucial for maintaining microenvironmental homeostasis (36, 50). During aging, there is a reduction in the expansion of lymphatic vessels, and the production of Myh11+ cells diminish following genotoxic stress (9) (**Figure 2**). Moreover, aged mouse bones show impaired upregulation of CXCL12 secretion from lymphatic endothelial cells post-irradiation, indicating that aging alters normal lymphangiocrine functions. Additionally, markers of cellular senescence, such as *p16* and *p27*, are increased in the lymphatic endothelial cells of aged mice, while proliferation markers *Ki67* and lymphatic endothelial marker *Vegfr3* are downregulated. These alterations suggest that aging profoundly impacts the functionality of lymphatic endothelial cells (9).

Overall, the interplay between aging, inflammation, lymphatic function, and musculoskeletal health underscores the importance of understanding these mechanisms to develop therapeutic strategies aimed at mitigating bone loss and improving outcomes in aging populations.

## Dysregulated lymphatic vessels in musculoskeletal diseases

Bone marrow edema is characterized by pain and increased interstitial fluid within the bone marrow. This condition is associated with various clinical features, including fractures, trauma, OA, RA, ischemic lesions, and some infectious lesions (53). Both current and historical studies suggest that lymphatics may also play a vital role in drainage functions within the bone (9, 28). Further investigations are needed to elucidate the mechanisms underlying the contraction of bone lymphatic vessels.

The dysregulation of lymphatic vessels significantly affects the musculoskeletal system, with poor mobility frequently observed in patients with lymphedema. Individuals afflicted with chronic lymphedema often develop secondary musculoskeletal diseases; however, studies linking lymphedema with skeletal disorders remain limited. Edema in damaged tissues can persist, resulting in pain, stiffness, and decreased function among patients living with musculoskeletal disorders (54). One study noted that lymphedema impairs fracture recovery in rats, although its association with human recovery remains unclear (55). Additionally, lymphedema has been correlated with dual-energy X-ray absorptiometry (DXA) measurements of bone mineral density in the limbs of patients with breast cancer (56).

Lymphatic endothelial cells secrete lymphangiocrine signals, including chemokines, which facilitate the migration of various leukocyte subtypes during inflammation. For instance, the chemokine CCL21 produced by lymphatic endothelial cells interacts with dendritic cells (DCs), T cells, and macrophages through its receptor CCR7, thereby influencing immune responses at the inflammation site (57–59). In the context of myocardial infarction, lymphatic endothelial cells expressing S1pr1 enhance macrophage clearance, highlighting their role in tissue repair (60).

Lymphatic vessel expansion in bone has often been viewed as a pathological condition, exemplified by complex lymphatic anomalies (CLA). In contrast to injury-induced lymphatic vessel expansion that promotes regeneration in normal bones, CLA is characterized by inappropriate lymphangiogenesis and gradual bone loss (61). Conditions associated with CLA include Kaposifirm lymphangiomatosis (KLA), generalized lymphatic anomaly (GLA), and Gorham-Stout Disease (GSD), with the pathophysiology of these disorders being closely linked to specific somatic gene mutations. For instance, activated somatic mutations in *KRAS* lead to GSD (62, 63), while mutations in *PIK3CA* and *NRAS* are implicated in GLA (64–66). KLA is a rare, aggressive subtype of CLA marked by multifocal infiltrative lymphatic proliferation with somatic NRAS Q61R mutations leading to constitutive activation of PI3K/AKT/mTOR and MAPK/ERK pathways (67, 68). In GSD (**Figure 2**), multiple mechanisms have been identified that contribute to lymphatic expansion and subsequent bone loss. The hyperactivating somatic mutation in KRAS triggers hyperactivation of the RAS-MEK signaling pathway in lymphatic endothelial cells, resulting in lymphatic malformations (63). Additionally, increased PI3K signaling in lymphatic endothelial cells, and overexpression of VEGF-C by bone cells, promotes lymphatic formation in bone and is associated with bone loss in transgenic mouse models (32, 64). Elevated VEGF-C levels have also been detected in the serum of individuals with GSD (69). VEGF-C is known to act as a target gene for RANKL in osteoclasts, enhancing osteoclastic bone resorption through an autocrine mechanism (70). Furthermore, lymphatic endothelial cells encourage osteoclast differentiation in mouse models via the expression of macrophage colony-stimulating factor (71). Together with RANKL, these factors are essential for osteoclastogenesis (71, 72). The increased presence of osteoclasts in GSD lesions suggests that activation of osteoclastogenesis may significantly contribute to osteolysis (73).

Current therapies for kaposiform lymphangiomatosis (KLA) and central conducting lymphatic anomaly (CLA) remain limited and largely palliative. Sirolimus yields partial responses in approximately 58% of patients but is constrained by adverse effects, including immunosuppression and hyperlipidemia. Trametinib has shown anecdotal pain relief yet lacks robust clinical trial data and is often ineffective in patients harboring NRAS mutations due to resistance. Sclerotherapy may reduce cystic lesions but is ineffective for solid, infiltrative disease and has a high recurrence rate. The development of direct NRAS inhibitors and rational combination therapies is urgently needed. Emerging evidence suggests that elevated serum VEGF-C levels may serve as a biomarker of sirolimus resistance, supporting a precision medicine approach. Additionally, anti-IL-6 agents such as tocilizumab show preclinical promise in mitigating osteolysis (67), highlighting the potential of immunomodulation as a therapeutic strategy.

### Osteoarthritis

**Figure 3:**
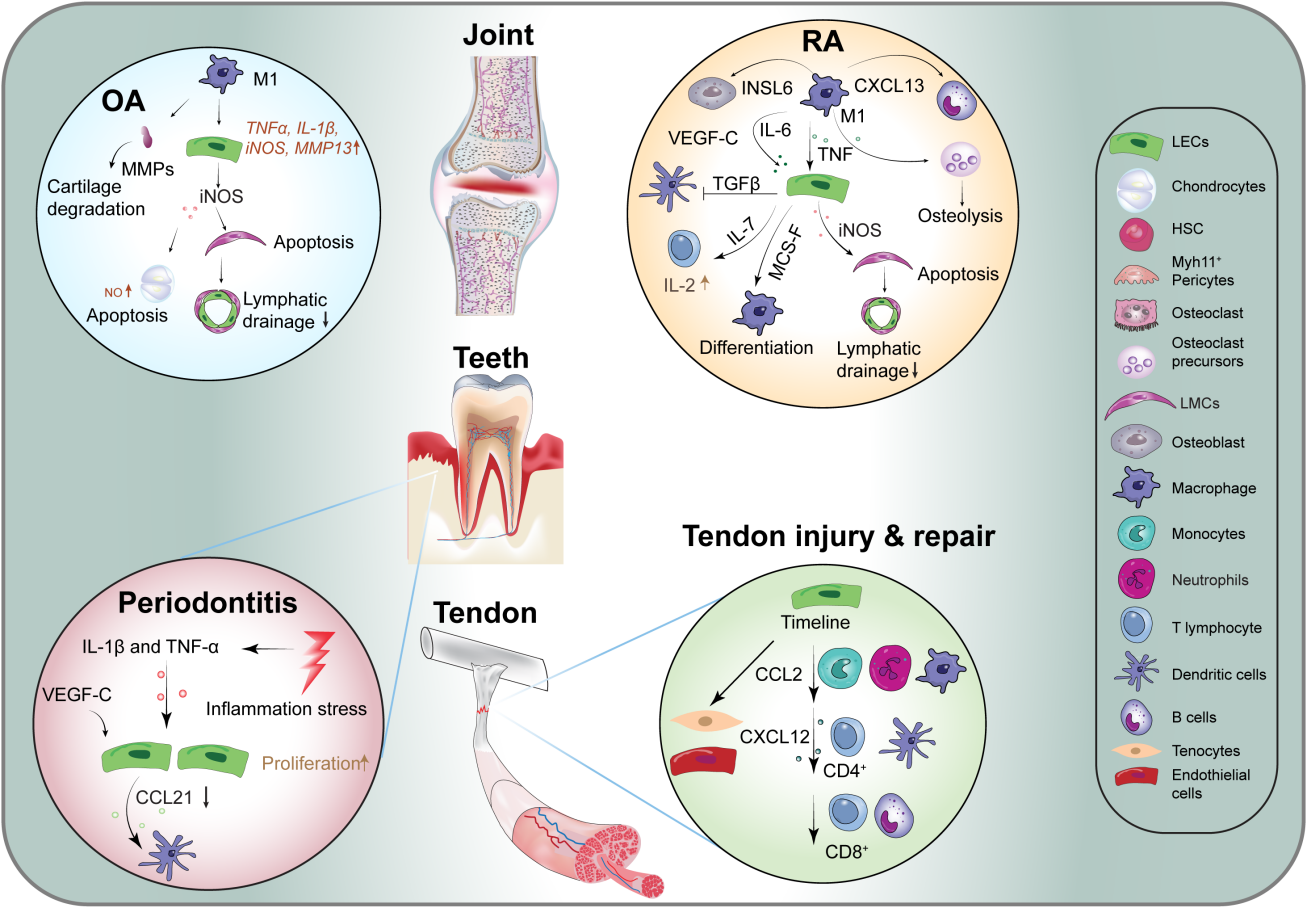
Lymphangiocrine signaling in various diseases. CCL2, C-C Motif Ligand 2; CXCL12, C-X-C Motif Chemokine Ligand 12; CXCL13, C-X-C Motif Chemokine Ligand 13; CXCL21, C-X-C Motif Chemokine Ligand 21; iNOS, Induced Nitric Oxide Synthase; INSL6, Insulin Like 6; M-SCF, Macrophage Colony Stimulating Factor; MMPs, Matrix Metalloproteinases; OA, Osteoarthritis; RA, Rheumatoid Arthritis; Sem3a, Semaphorin-3a; TNF, Tumor Necrosis Factor; VEGF-C, Vascular Endothelial Growth Factor -C; ILs, Inflammatory Factors.

OA is a chronic, progressive degenerative joint disease that impacts multiple components of the joint, including articular cartilage, subchondral bone, ligaments, joint capsule, and synovium. Characterized by cartilage degradation, relatively mild synovial inflammation, and subchondral bone destruction, OA represents a significant cause of disability (74). Lymphatic vessels are involved actively in the pathology of OA (**Figure 3**). For instance, a mouse model of OA induced by meniscal ligament injury demonstrated increased lymphatic vessel density under mild conditions, while a significant reduction in lymphatic vessel density and lymphatic clearance were observed in more severe cases (17). Similarly, patients with advanced OA exhibited a marked decrease in lymphatic vessel density (17).

In OA-affected joints, there is an increase in the number of M1 macrophages, a phenomenon driven by pro-inflammatory signals (75). These M1 macrophages enhance the expression of various inflammatory genes in lymphatic endothelial cells, including *TNFα*, *IL-1β*, inducible nitric oxide synthase (*iNOS*), and matrix metalloproteinase-1 (*MMP1*) (76). The inflammatory cytokine TNFα promotes the secretion of iNOS from lymphatic endothelial cells, leading to chondrocyte apoptosis via the production of nitric oxide (NO) (77, 78). iNOS has also been implicated in the apoptosis of lymphatic smooth muscle cells, which subsequently impairs lymphatic drainage function (79). Moreover, MMPs, which are enzymes responsible for degrading cartilage, are expressed at elevated levels in arthritic joints (80). The impaired drainage function of synovial lymphatic vessels prevents the effective removal of catabolic large molecules, exacerbating the damage associated with arthritis.

Aging is a primary risk factor for the development of OA (74). Studies involving aged mice have shown significant impairments in joint clearance, reduced synovial inflow, and compromised lymph node drainage capacity (81). These changes are associated with decreased expression of VEGF-C in the synovium of aged mice (81). Treatment with VEGF-C has been shown to improve synovial lymphatic function in aged mice (81). Thus, targeting synovial lymphatics and modulating VEGF-C/VEGFR-3 signaling pathways could represent a promising therapeutic strategy for the treatment of OA.

### Dental disease

Dental pulp, the soft tissue located at the center of the tooth, exhibits inconsistent perspectives regarding the existence of lymphatic vessels across different species (82, 83). Dental diseases, particularly periodontitis, primarily arise from bacterial invasion of the gingiva, which induces inflammation and subsequent alveolar bone loss (84). Despite the utilization of advanced techniques to confirm the presence of lymphatic vessels in dental pulp (82, 83, 85), the link between lymphatic vessel formation and the degree of inflammation remains poorly understood (86). Studies using a transgenic mouse model expressing the K14-VEGF receptor 3-Ig (K14) have illuminated the critical role of lymphatic vessels in the gingiva, highlighting their importance in maintaining capillary fluid balance and protecting against bone loss in response to *Porphyromonas gingivalis* infection (87, 88) (**Figure 3**). In particular, transgenic mice exhibited greater bone loss compared to wild-type counterparts, alongside significantly elevated periodontal levels of pro-inflammatory cytokines, including IFN-γ, IL-1β, and G-CSF (87).

During the progression of periodontitis, human gingival epithelial cells respond to P. gingivalis lipopolysaccharide (LPS) by expressing VEGF-C (89). An increase in VEGF-C expression is observed in epithelial keratinocytes, accompanied by an elevated presence of CD45+ immune cells that also express VEGF-C (90). Additionally, both IL-1β and TNF-α, recognized as potent stimulators of lymphangiogenesis, are upregulated (90). The cytokine CCL21 is present in lymphatic endothelial cells and plays a role in recruiting lymphocytes and mature DCs via CCR7 (91). Notably, the expression of CCL21 in human gingival lymphatic vessels is downregulated during periodontitis (91), a trend also observed in mice with periodontitis (92).

In the context of dental aging, an increase in LYVE1-labeled lymphatic capillaries devoid of smooth muscle coverage has been identified in aged dental pulp (85). This observation suggests that the lack of smooth muscle contraction hinders efficient lymph fluid drainage in older pulp tissue. Moreover, dilated capillary lymphatic vessels are noted in aged pulp, indicating a pronounced inflammatory response and compromised lymphatic drainage (85). Consequently, aging adversely impacts lymphatic function in dental tissues, leading to exacerbated inflammatory responses and impaired clearance of lymphatic fluid.

### Rheumatoid arthritis

RA is a prevalent autoimmune disease primarily affecting the joints, with an estimated prevalence of 0.5-1% in the population. RA imposes significant health and economic burdens on both patients and society (93). The hallmark of RA is synovitis, which refers to the inflammation of the synovial membrane. This condition leads to symptoms such as redness, warmth, swelling, severe pain, and stiffness in the affected joints, driven by increased vascularization and immune cell infiltration. Additionally, the deterioration of articular cartilage and the underlying bone is a critical feature of RA, ultimately restricting joint mobility and leading to complete joint destruction (94, 95).

Recent studies have highlighted the role of lymphangiogenesis within the synovium and the expansion of draining lymph nodes in chronic arthritis, as well as lymphatic dysfunction associated with lymph nodes collapse in RA patients (96–99) (**Figure 3**). Impaired lymphatic drainage in the hands of RA patients with active disease has been observed compared to healthy controls. Although the pathogenesis of RA remains complex and not fully elucidated, the lymphatic immune microenvironment plays a vital role in immune surveillance and maintaining interstitial fluid balance, which are crucial in the development and progression of RA (96, 100). These observations elucidate the exacerbation of synovitis and joint damage, particularly due to the accumulation and retention of inflammatory cells and factors in the joints of RA patients, resulting from impaired efferent lymphatic flow (98, 101). Experimental models of RA indicate that lymphatics are responsible for clearing catabolic factors, cytokines, and inflammatory cells, including macrophages and monocytes, from the inflamed synovium, thus identifying lymphatics as potential therapeutic targets (96, 102). For instance, CCL19 is expressed by lymphatic endothelial cells, while CCL21 levels are upregulated in RA fibroblasts and macrophages (103, 104). The presence of CCL21 is implicated in the induction of erosive arthritis through the recruitment of CCR7+ monocytes, promoting M1/Th17 polarization, osteoclastogenesis, and neovascularization (105).

In the context of RA, increased levels of VEGF-C have been detected in the synovial fluid of affected patients (106). Within the inflammatory microenvironment, Prox1 is activated via the nuclear factor-κB (NF-κB) signaling pathway (107), enhancing the promoter activity of VEGFR-3. This process amplifies receptor expression on lymphatic endothelial cells and heightens susceptibility to VEGF-C, resulting in increased lymphatic proliferation, survival, and migration, thereby promoting lymphatic vessel remodeling (99). Additionally, VEGF-C expression is elevated in CD11b+/Gr-1− osteoclast precursors during inflammatory erosive arthritis (96). Research using animal models indicates that lymphatic drainage is augmented during the early stages of inflammatory-erosive arthritis; however, as inflammation progresses to a more chronic state, lymphatic clearance becomes compromised due to loss of lymphatic vessel contraction and collapse of draining LNs (97, 108, 109). This compromise is attributed to lymphatic endothelial cells producing iNOS in response to TNF in the inflamed joint (79, 110, 111). Moreover, in mouse models of RA, TNFα-polarized macrophages are known to influence osteoblast differentiation through the secretion of insulin-like 6 peptides (112). In TNF-transgenic mice exhibiting advanced arthritis, anti-TNF therapy significantly reduces synovitis and restores lymphatic contraction. This restoration is associated with enhanced cellular egress from the joint, facilitated by active lymphatic transport through the draining LNs. These findings underscore the therapeutic potential of anti-TNF therapy in treating RA and its positive impact on lymphatic function (102, 113, 114).

It is noteworthy that the structure and cellular composition of LNs in RA patients undergo significant changes (96, 110), suggesting that lymphatic vessels and nodes may serve as promising biomarkers of disease activity and prognosis in RA. The popliteal LN has been identified as a biomarker, evidenced by its swelling and contrast enhancement during prolonged asymptomatic phases, preceding symptomatic stages, at which point it collapses alongside the erosive changes in synovitis, cartilage, and bone (96, 108). This collapse of the popliteal LN correlates with the translocation of B cells from the follicles to the sinuses, leading to obstruction of the lymphatic sinuses. Consequently, therapies aimed at B cell depletion may mitigate arthritic flare-ups by eliminating obstructive B cells and restoring lymphatic drainage in inflamed joints (99, 109). Taken together, targeting lymphatic function presents a promising strategy for managing RA in the future. However, further investigation is necessary to elucidate the role of lymphangiocrine signaling in the pathology of RA.

### Tendon injury, and heterotopic ossification

Tendons and ligaments are specialized types of fibrous connective tissue that connect muscles to bones and bones to other bones, respectively. However, there is limited information available regarding the presence and role of lymphatic vessels in ligaments. In studies examining the posterior cruciate ligament, lymphatic vessels have been identified within the loose connective tissue interspersed among collagen fibers (115). Nonetheless, specific immunohistochemical staining techniques are necessary to confirm their presence definitively (116).

Following acute Achilles tenotomy, both adaptive and innate immune cells respond within the tendons and LNs to facilitate tendon repair (**Figure 3**). M1 macrophages accumulate at the injury site within the first week, while the number of M2 macrophages increases during the second week post-injury (117). Additionally, there is an upregulation of the monocyte chemoattractant *Cxcl12*, which promotes the trafficking of various immune cell populations, including T cells and B cells, in the ensuing weeks following the injury (118).

Though it is well recognized that tendons are poorly vascularized compared to other tissues, they are surrounded by lymphatic vessels (119). The importance of lymphatic vessels in tendon disease has often been underestimated. In a rat model of Achilles tendon injury, an expansion of the lymphatic endothelium at the injury site was observed (120, 121). Analysis of lymphatic vessels, characterized by markers such as CD31, PDPN, and LYVE1 around the Achilles tendons of ScxGFP mice, reveals close interactions with tenocytes and blood vessels. Furthermore, hypertrophy of the LNs and the presence of plasma cells indicate that the draining LNs are actively responding to tendon injury and repair (118). Additional studies are warranted to further elucidate the role of lymphatic vessels in tendon healing (122).

Heterotopic ossification (HO) is characterized by abnormal bone formation in soft tissue and muscle outside of the skeletal system (123, 124). This condition can arise from genetic factors, as seen in fibrodysplasia ossificans progressiva, or it may be acquired following trauma, referred to as trauma-induced HO (125). Acquired HO is more commonly observed and typically results from significant trauma, burns, surgeries, or central nervous system injuries (126). The progression of acquired HO involves several distinct stages, including inflammation, chondrogenesis, osteogenesis, and maturation.

Injury can also prompt HO to develop in tendons (125), with lymphatic endothelial cells playing a significant role in the pathological progression of HO (121, 127). At the site of tenotomy, lymphatic endothelial cells accumulate in areas rich in fibroblasts and chondrocytes, which express VE-Cadherin and Sox9, contributing to HO development (121, 128). Furthermore, the absence of fibroblast growth factor receptor 3 expression in lymphatic endothelial cells hinders lymphangiogenesis via a BMPR1a-pSmad1/5-dependent mechanism. This impairment of local lymphatic drainage, alongside persistent high levels of inflammation, exacerbates the progression of HO (127). Moreover, LNs are implicated in musculoskeletal repair and the development of HO, aiding in the formation and maturation of bone-cartilage stromal progenitor cells into fully developed HO (121). Additionally, in cases of traumatic HO, injury stimulates mesenchymal progenitor cells expressing VEGF-C and tenocytes, promoting lymphangiogenesis at the injury site and facilitating bone tissue formation (128). While the involvement of lymphatic endothelial cells in HO is well supported by current literature, real-time *in vivo* studies are necessary to fully elucidate the underlying mechanisms.

## Sex differences in lymphatics

While endothelial cells in the blood vascular system exhibit well-characterized sexually dimorphic phenotypes, sex-specific differences in the lymphatic system—particularly in the context of musculoskeletal health—remain less well understood (129). Females are disproportionately affected by lymphatic disorders such as primary lymphedema, lipedema, and lymphangioleiomyomatosis (LAM) (130–132). These conditions often coincide with hormonal fluctuations during puberty, pregnancy, and menopause, suggesting that lymphatic function and dysfunction are hormonally regulated. Estrogen receptor is expressed on lymphatic endothelial cells, indicating that these hormones may directly influence lymphatic vessel behavior (133). For instance, estrogen has been shown to promote lymphangiogenesis and modulate the expression of key lymphatic markers such as VEGFR-3 and LYVE-1, both of which are essential for lymphatic development and function (134). Sex-based differences in lymphatic function may contribute to the observed disparities in musculoskeletal conditions between males and females. For example, women are more prone to osteoarthritis and rheumatoid arthritis, both inflammatory conditions marked by joint degradation, where impaired lymphatic drainage may exacerbate disease progression (51, 101, 135). Additionally, estrogen’s influence on lymphatic vessels could affect bone remodeling processes, potentially contributing to differences in bone density and strength between the sexes (133, 136). Further research into sex-specific lymphatic biology—particularly as it relates to musculoskeletal health—is urgently needed. Understanding how sex hormones modulate lymphatic function could pave the way for targeted therapies that address lymphatic-related musculoskeletal disorders and improve outcomes, especially for conditions that disproportionately affect women.

## Therapeutic potential and future perspectives

Recent studies examining bone tissue have emphasized the role of lymphangiocrine signaling in bone-immune communication, establishing it as a promising target for therapeutic intervention due to its manipulability (137). While clinical attempts to target VEGF-C have yet to yield successful results, with drugs such as VGX-100 and OPT-302 being the only agents developed for targeting human VEGF-C, neither has gained approval (81, 138, 139). Thus, exploring new therapeutic targets in lymphangiocrine signaling is both timely and warranted.

In experimental murine models, transferring lymphatic endothelial cells from young to older mice via intratibial injections has been shown to enhance immune responses, improve lymphangiocrine signaling, and promote lymphangiogenesis following stress (9). These findings collectively suggest that targeting lymphangiocrine signaling could be a viable strategy for treating various musculoskeletal disorders and diseases. However, several critical points must be addressed before translating this novel concept into clinical therapies. Firstly, the mechanisms by which lymphatic vessels in bone mediate immune responses remain unclear, particularly their roles in bone infections, inflammation, and autoimmune diseases affecting bone health. Despite recent studies elucidating the novel roles of lymphatic vessels in bone biology, the mechanisms driving immune responses related to musculoskeletal diseases are not fully understood. Secondly, the functional capacity of lymphatic vessels in bones is still not clear, and the precise mechanisms controlling lymphatic flow within the bone are yet to be determined, including factors such as exercise, injury, or aging that may influence this flow. Additionally, it is crucial to investigate the full extent of the lymphatic network in bone, particularly the different types of lymphatic vessels associated with various regions and functions within the bone structure. Furthermore, it is essential to identify the signaling molecules produced by bone lymphatics and to understand how these molecules interact with other cell types within the bone microenvironment. Future research should concentrate on elucidating specific signaling pathways and their effects on key cell populations, such as osteoblasts, chondrocytes, muscle cells, and fibroblasts.

The involvement of lymphatic vessels in bone health, repair, and remodeling represents an intriguing area for ongoing investigation. Further research is required to clarify the specific contributions of lymphatics to bone biology and to assess their potential role in bone-related diseases, including osteoporosis and fracture healing. Additionally, understanding how musculoskeletal lymphatics contribute to immune responses during tissue injury or infection is essential. Future studies should explore the interactions between lymphatic vessels, immune cells, and the outcomes of musculoskeletal infections and inflammatory conditions. Advancements in imaging techniques are imperative for comprehensive *in vivo* visualization and investigation of musculoskeletal lymphatics. Progress in this area will enhance our understanding of the structure and function of lymphatics within these tissues. Moreover, examining the impact of inflammation on musculoskeletal lymphatics and exploring potential modulation of these pathways for therapeutic purposes represents an exciting avenue for research. Future investigations should focus on the potential of targeting lymphatic pathways to mitigate inflammatory responses and facilitate tissue healing, particularly concerning musculoskeletal conditions such as arthritis and soft tissue injuries.

Translating our increasing understanding of musculoskeletal lymphatics into clinical applications presents a promising frontier, offering the potential to develop innovative treatments for musculoskeletal disorders and injuries. Ongoing research efforts should prioritize the development and refinement of these therapeutic approaches, ensuring that fundamental science findings translate effectively to clinical practice. Ultimately, the study of musculoskeletal lymphatics stands at the threshold of revealing novel insights into tissue health and disease. Addressing the questions posed in this review, along with pursuing the outlined future perspectives, will undoubtedly enhance our comprehension of musculoskeletal lymphatics, foster innovative solutions, and improve patient outcomes in the field of musculoskeletal medicine.
